# Quantitative and qualitative differences in celiac disease epitopes among durum wheat varieties identified through deep RNA-amplicon sequencing

**DOI:** 10.1186/1471-2164-14-905

**Published:** 2013-12-19

**Authors:** Elma MJ Salentijn, Danny G Esselink, Svetlana V Goryunova, Ingrid M van der Meer, Luud JWJ Gilissen, Marinus JM Smulders

**Affiliations:** 1 Plant Research International, Wageningen UR, P.O. Box 16, Wageningen, AA NL-6700, The Netherlands; 2 Vavilov Institute of General Genetics, Russian Academy of Sciences, Moscow 119991, Russia

## Abstract

**Background:**

Wheat gluten is important for the industrial quality of bread wheat (*Triticum aestivum* L*.*) and durum wheat (*T. turgidum* L.). Gluten proteins are also the source of immunogenic peptides that can trigger a T cell reaction in celiac disease (CD) patients, leading to inflammatory responses in the small intestine. Various peptides with three major T cell epitopes involved in CD are derived from alpha-gliadin fraction of gluten. Alpha-gliadins are encoded by a large multigene family and amino acid variation in the CD epitopes is known to influence the immunogenicity of individual gene family members. Current commercial methods of gluten detection are unable to distinguish between immunogenic and non-immunogenic CD epitope variants and thus to accurately quantify the overall CD epitope load of a given wheat variety. Such quantification is indispensable for correct selection of wheat varieties with low potential to cause CD.

**Results:**

A 454 RNA-amplicon sequencing method was developed for alpha-gliadin transcripts encompassing the three major CD epitopes and their variants. The method was used to screen developing grains on plants of 61 different durum wheat cultivars and accessions. A dedicated sequence analysis pipeline returned a total of 304 unique alpha-gliadin transcripts, corresponding to a total of 171 ‘unique deduced protein fragments’ of alpha-gliadins. The numbers of these fragments obtained in each plant were used to calculate quantitative and quantitative differences between the CD epitopes expressed in the endosperm of these wheat plants. A few plants showed a lower fraction of CD epitope-encoding alpha-gliadin transcripts, but none were free of CD epitopes.

**Conclusions:**

The dedicated 454 RNA-amplicon sequencing method enables 1) the grouping of wheat plants according to the genetic variation in alpha-gliadin transcripts, and 2) the screening for plants which are potentially less CD-immunogenic. The resulting alpha-gliadin sequence database will be useful as a reference in proteomics analysis regarding the immunogenic potential of mature wheat grains.

## Background

Wheat-containing products are worldwide an important part of the human daily menu. Hexaploid bread wheat (*T. aestivum* L., ABD genomes) and tetraploid durum wheat (*T. turgidum* L*.*, AB genomes) are the most common wheat species grown for food production. The differences in food-technological qualities between both wheat species are largely determined by the composition of the gluten fraction in the grains. Gluten, the water-insoluble fraction of wheat seed-storage proteins, consists of the high- and low molecular weight subunit glutenins (HMW-GS and LMW-GS) and the monomeric gliadins (α/β-, γ- and ω-gliadins) [1].

Gluten proteins are relatively resistant to proteolysis. Several specific bioactive gluten peptides have been identified that survive proteolysis in the human intestine and that can stimulate T cells [2-5] and trigger celiac disease (CD) in genetically susceptible individuals. CD is a T cell mediated chronic inflammatory condition of the small intestine [5,6] with prevalence between 0.5 and 2% in human populations [7,8]. The immunogenic peptide sequences have highly specific cores of at least nine amino acids length [5,9], and become active after deamidation by the enzyme tissue transglutaminase in the intestine [10]. There are natural epitope variants that lack immunogenicity due to single or multiple amino acid substitutions. For instance, a P to S substitution at the epitope core position 8 was shown to be sufficient to abolish T cell stimulation [11].

T cell clones isolated from intestinal celiac lesions showed differential responses to diploid *Aegilops* and *Triticum* species that are related to the ancestors of the A, B, and D genomes [12-14]. These differences in T cell responses between diploid *Aegilops* and *Triticum* species especially related to the presence of three CD epitopes derived from alpha-gliadins, DQ2.5-Glia-α1 (PFPQPELPY), DQ2.5-Glia-α2 (PQPELPYPQ) and DQ2.5-Glia-α3 (FRPEQPYPQ); in these epitopes glutamic acid (E) is originating from deamidated glutamine (Q) [5,9,15,16]. The source of these epitopes, the alpha-gliadins, are encoded by a multigene family located on three homoeologous loci, *Gli-A2*, *Gli-B2* and *Gli-D2* on the short arms of wheat group 6 chromosomes (6AS, 6BS and 6DS). Estimates of the copy number of alpha-gliadins range from 25 copies to even 150 copies per haploid genome, reflecting the large complexity of this gene family [17-19]. The large majority (up to 87% in hexaploid wheat) of the genes contain internal stop codons and are presumably pseudogenes [20,21].

Limiting the abundance of CD epitopes in food products may reduce the risk of sensitization of the immune system of the group of people that are genetically susceptible for CD. In order to breed and select for wheat varieties with significantly reduced immunogenic potential to cause CD it is necessary to accurately estimate the quantity and quality of the CD epitope load in gluten. Up to now, the ability for high throughput quantification of CD epitopes by presently available assays based on T cell clones and on monoclonal antibodies is very limited, mainly because of the high complexity of the wheat material on the one hand, and the laboriousness of *in vitro* T cell assays and the promiscuity of the monoclonal antibodies on the other hand [22,23]. In addition, most commercial kits with monoclonal antibodies detect gluten, not CD epitopes.

Next-generation sequencing platforms offer now the possibility of efficient and accurate deep sequencing of genetic variation at moderate costs [24,25]. Still, the application of such technologies in bread wheat is a big challenge due to the large genome (17 Gbp, five times the size of the human genome), the allohexaploid nature and the abundance of repetitive sequences [26]. To reduce difficulties with the alignment of sequences for the detection of single nucleotide polymorphisms (SNPs), often ‘reduced representation libraries’ are used that include only a subset of sequences from several individuals representative for different populations [27,28] or from tissue-specific transcriptomes (RNAseq) [29]. In wheat, next-generation sequence studies for SNP detection have been performed on material with a reduced complexity such as the bread wheat transcriptome [30,31], diploid *Aegilops tauschii*[32], or specific subsets of DNA fragments [33].

RNAseq by Illumina sequencing produces short sequences. Short reads of alpha-gliadins cannot readily be assembled as the members of the gene family are very similar. Hence, such a method would enable to calculate average presence/absence of epitopes, but not show how these epitopes are distributed across genes. Quantitative PCR methods require the design of specific primers for the amplification of specific family members. It can be done in gene families when the members are sufficiently differentiated (e.g., gene-specific primer pairs were designed for each of the 31 Mal d 1 genes in apple, some of which cause apple allergy [34]), but the alpha-gliadins are far too similar and too numerous to enable development of primers that would allow quantitative amplification of all members.

With the aim to develop a pre-screening tool for the classification of wheat varieties according to their CD immunogenic potential, here a next generation sequencing technology was developed and applied that uses a 454 sequencer to perform RNA-amplicon sequencing. The 454 reads are sufficiently long to enable direct sequencing of the region of alpha-gliadin genes, that includes the three major CD epitopes. The complexity of the alpha-gliadin gene family was reduced by (a) focussing on the N-terminal, CD epitope containing region of alpha-gliadins, and (b) avoiding silent pseudogenes by sequencing the alpha-gliadin transcriptome (cDNA) of developing seeds. The method is applicable to wheat species regardless of ploidy level. A custom 454 sequence analysis pipeline was used to quantify CD epitopes and their variants in the alpha-gliadin transcriptomes of a set of 77 individual plants from 61 different durum wheat accessions, by determining the normalised transcript abundances for the respective CD epitopes and variants thereof.

## Results

### RNA-amplicon sequencing and sequence analysis pipeline

**Figure 1:**
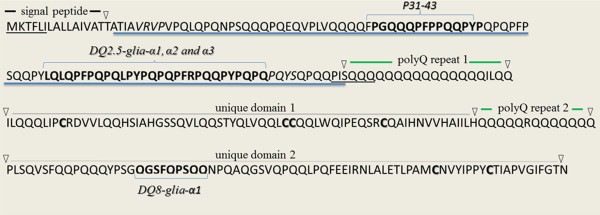
**Amino acid sequence of a typical alpha-gliadin.** Amino acid sequence of a typical alpha-gliadin (gi|289718578|gb|ADD17012.1|) and location of CD epitopes. Consecutively the signal peptide, repetitive domain (blue undelined), polyglutamine repeat 1, unique domain 1, polyglutamine repeat 2 and unique domain 2 are shown (according to Anderson and Greene [20]). In bold, conserved cysteins; underlined, location of PCR primers; in italics, motifs for sequence trimming.

**Figure 2:**
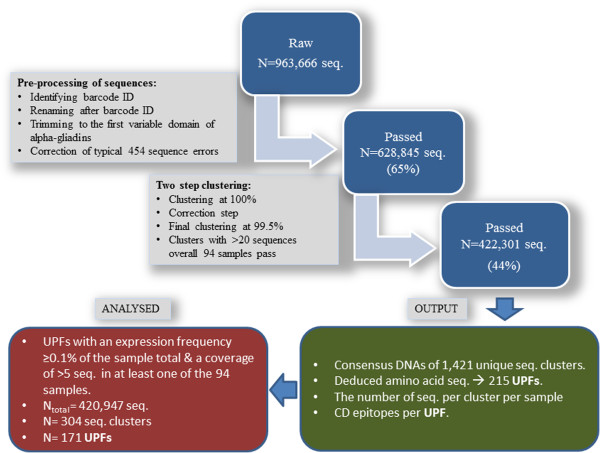
Overview of the sequence analysis pipeline.

To assess a large, diverse set of durum wheat landraces and genebank accessions for their CD epitope content, a deep 454 RNA-amplicon sequencing pipeline was developed to target the genetic variation in the first repetitive domain of alpha-gliadins (Figure 1, underlined in blue), which contains the major CD epitopes DQ2.5-glia-α1, DQ2.5-glia-α2 and DQ2.5-glia-α3. A custom sequence analysis pipeline (Figure 2) was developed to process the 454-reads derived from the RNA-amplicon and subsequently the variation in the abundance of unique alpha-gliadin transcripts was determined. In this way an estimate of the alpha-gliadin protein composition and CD epitope composition was made for the 77 durum wheat plants from 61 different durum wheat cultivars and accessions, including landraces and breeders material (Additional file 1: Table S1).

**Figure 3:**
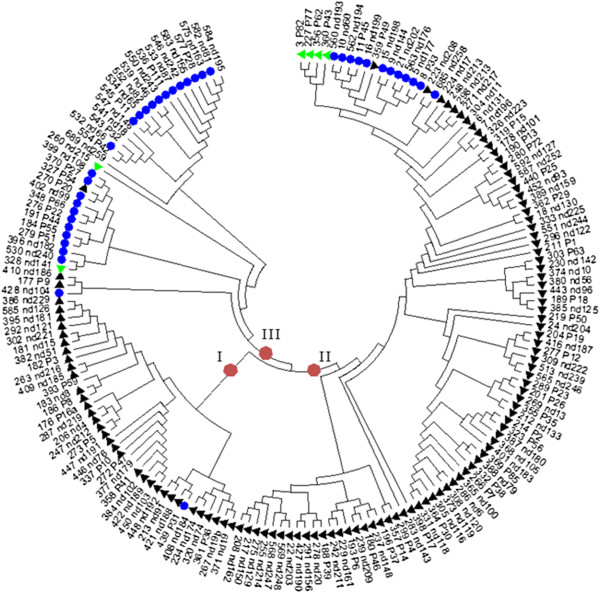
**Neighbor-joining topology tree of alpha-gliadins.** Neighbor-joining topology tree of alpha-gliadins (unique deduced protein fragments, UPFs). I, II and III in red circles = NJ-topology groups. Green triangles = signature of the D sub-genome; black triangle = signature of the A sub-genome; blue circles = signature of sub-genome B.

**Figure 4:**
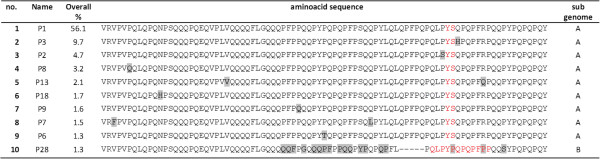
**Alignment of the ten most abundant unique alpha-gliadin fragments.** The amino acid sequences of the ten overall most abundant unique alpha-gliadin protein fragments (repetitive domain; UPFs) with in red the motifs that are indicative for a specific sub-genome of wheat. In grey, amino acid variation compared to UPF-P1. Overall% = normalised transcript abundances overall 94 samples

For 454 RNA-amplicon sequencing, alpha-gliadin amplicons derived from cDNAs from developing seeds of single plants were uniquely labelled with a 10 bp identification (ID) sequence and subsequently sequenced in three 454 runs to obtain detailed sequence information on the expressed alpha-gliadin fraction in developing seeds (Table 1). In total over all samples 420,947 454-reads derived from alpha-gliadin transcripts were analysed (average 4,478 reads per plant, 240–281 bp in size after trimming). In the process of sequence analysis these reads were organized into 304 ‘unique sequence clusters’ (average 68 clusters per plant) that represented the CD epitope-containing regions of alpha-gliadin genes expressed in the developing seeds. Based on the nucleotide sequences of these unique sequence clusters, 171 ‘unique deduced protein fragments’ (UPFs) were predicted (average 50 UPFs per plant) that represented the predicted amino acid variants of the first variable domain of alpha-gliadins that are expressed in the endosperm. A UPF could be encoded by one or more sequence clusters. In total 116 of the 171 UPFs were encoded by a single unique sequence cluster. On the other hand one UPF (UPF-P1) was encoded by as many as 34 sequence clusters and this was the only UPF that was present in all 77 plants. The sequence variation among the UPFs was studied in a neighbour joining analysis and three groups were recognised (Figure 3). Based on the presence of several amino acid motifs (Table 2) that are specific for the different sub-genomes of wheat, the UPFs were assigned to a specific sub-genome. Although durum wheat contains only the A and B genome, six protein fragments with a D-genomic signature were found. Four of these came from a genebank accession of a landrace (CGN08360, ‘Diha Dzhavakhetskaja’) that is known to consist of a mixture of tetraploid and hexaploid genotypes with indistinguishable phenotypes [35]. Such a mixture is not uncommon in genebank accessions [35,36]. The two other alpha-gliadin protein fragments with a D genome signature showed also signatures of the B genome alpha-gliadins and were present at low abundance in several breeding lines of durum wheat (normalised transcript abundance 0.20 to 0.13). The great majority of the alpha-gliadin transcripts showed an A-genomic signature (Figure 4) with normalised transcript abundances for UPFs that ranged from 99 in the elite durum wheat variety ‘IXOs9442’, to 76 and 80 in ‘CIM-10204’, a line from the International Maize and Wheat Improvement Center (CIMMYT), and in line ‘CGN08006-2B’ respectively (Additional file 1: Table S1).

**Table 1:** 454 run statistics

**Run**	**F4SOYM002**	**GL4NQHJ02**	**G6WZP5402**	**Total**
N samples	23	24	48	95
Raw data	123,965	258,722	580,979	963,666
Passed reads	74,038	197,945	356,862	628,845
Contigs > 20 reads	241	269	911	1421
Length (min, median, max)	177, 261, 321	177, 261, 305	173, 262, 300	
Passed reads	42,162	158,054	222,085	422,301
% of raw reads	34%	61%	38%	
% of passed reads	57%	80%	62%	

**Table 2:** Sub-genome specific amino acid motifs

**Sub-genome specific motifs**	**Sub-genome**	**n**
Motif of two amino acids, ‘YS’	A	118
PQLPYL, PPQLPYP, LPQLPYP, QLPYPQPQPFPP	B	42
PQPQLPYPQ	D	4
PQPQLPYPQ + B sub-genome motif	D/B	2
No specific motif detected	Not assigned	5
	Total	171

Amino acid motifs in alpha-gliadin proteins that are specific for the different sub-genomes of wheat. n = number of unique alpha-gliadin protein fragments (UPFs) with the specific motif.

Each UPF contains three distinct loci for CD epitopes: DQ2.5-glia-α1, DQ2.5-glia-α2 and DQ2.5-glia-α3. Variants in these three loci found across all durum wheat samples are listed in Table 3 and Additional file 1: Table S1. They included several variants to the epitopes, among which four novel CD epitope variants (marked with a * in Table 3). The two novel variants of DQ2.5-glia-α1a, α1a-variant 4 and -5, and the novel variant of DQ2.5-glia-α3, were observed in respectively three-, two- and in a single plant at normalised transcript abundances of respectively maximally 10, 14 and 0.4. The other novel variant, DQ2.5-glia-α2 variant 4, was found in multiple accessions in varying abundances (ranging from 0.1 to 11).

**Table 3:** **The natural variation in CD epitopes in *T. turgidum***

**CD epitope name**	**Natural sequence variation**
**DQ2.5-glia-α1a**	**PFPQPQLPY**
**DQ2.5-glia-α1b**	**PYPQPQLPY**
α1a-variant 1	PF**L**QPQLPY
α1a-variant 2	PFPQPQL**S**Y
α1a-variant 3	PF**S**QPQLPY
α1a-variant 4	PFP**P**PQLPY*
α1a-variant 5	PFPQ**L**QLPY*
**DQ2.5-glia-α2**	**PQPQLPYPQ**
α2-variant 1	PQPQLPY**S**Q
α2-variant 2	**S**QPQLPY**S**Q
α2-variant 3	PQPQL**S**Y**S**Q
α2-variant 4	P**P**PQLPY**S**Q*
α2-variant 5	**L**QPQLPY**S**Q
α2-variant 6	F**P**PQLPYPQ
α2-variant 7	F**L**PQLPYPQ
**DQ2.5-glia-α3**	**FRPQQPYPQ**
α3-variant 1	F**P**PQQPYPQ
α3-variant 2	F**S**PQQPYPQ*
α3-variant 3	F**L**PQQPYPQ
α3-variant 4	F**PS**QQPYPQ
α3-variant 5	F**P**PQQ**S**YPQ
α3-variant 6	F**Q**PQQPYPQ
α3-variant 7	FRPQQ**S**YPQ

The natural sequence variation in HLA-DQ2.5 restricted T cell epitopes involved in CD (in their natural, non deamidated form); DQ2.5-glia-α1a and DQ2.5-glia-α1b, DQ2.5-glia-α2, DQ2.5-glia-α3, as present in cDNAs of *T. turgidum* accessions. Canonical CD epitope sequences in bold. * variants that have not previously been found in *T. aestivum*[11].

### Alpha gliadin expression profiles

**Figure 5:**
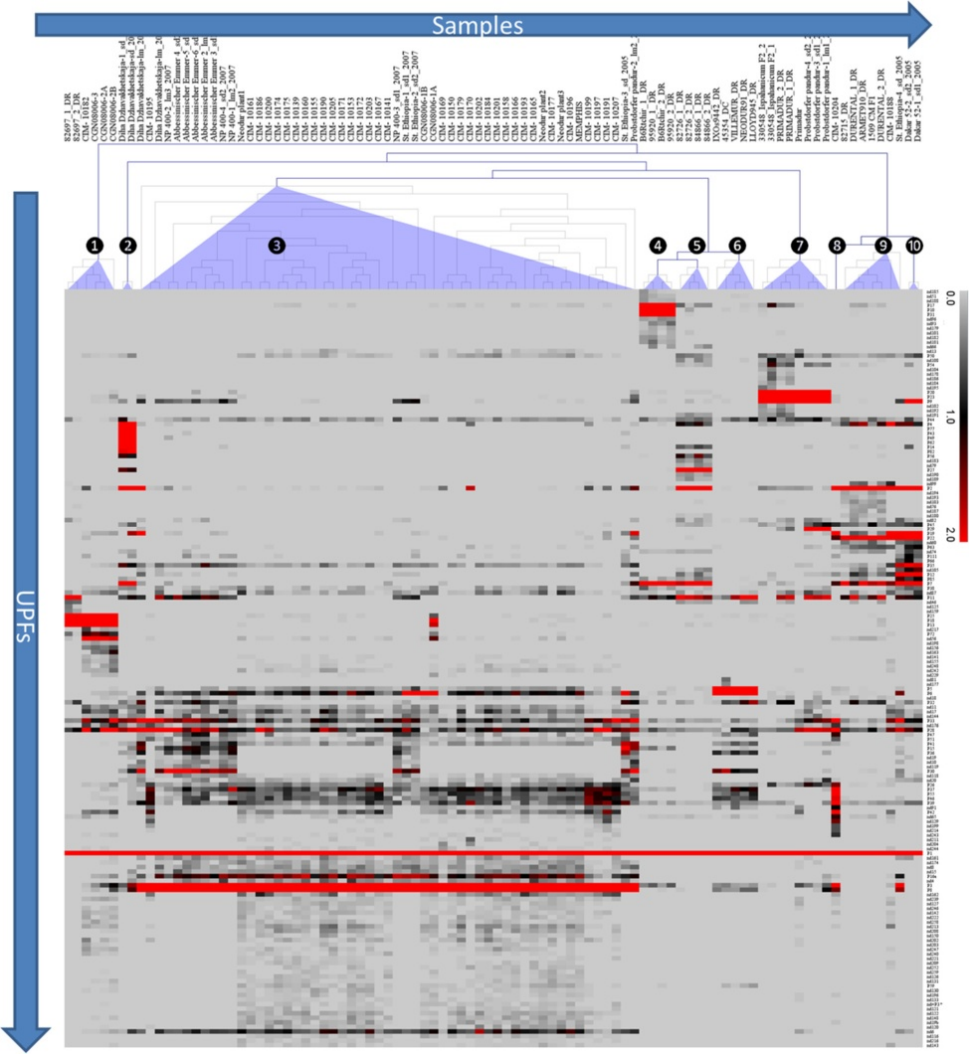
**Alpha-gliadin expression profiles of durum wheat plants.** The deduced unique alpha-gliadin protein fragments (UPFs) were differentially present in transcripts among the samples, at normalised transcript abundances ranging from zero (light grey) to 75.48 (2487/3295, for UPF- P1). The plants were clustered based on their UPF expression profiles using hierarchical clustering (average linkage groups, Pearson correlation). Heat map: normalised transcript abundances zero = light grey; normalised transcript abundance ~1 = black; normalised transcript abundance ~1.5 to > 2 = clear red.

Each 454 sample contained the alpha-gliadin fraction that is expressed in developing seeds of a single durum wheat plant. For each sample, a list of UPFs and epitope variants were obtained (qualitative output; Additional file 1: Table S1 and Additional file 2). The quantitative output of the sequence analysis pipeline consisted of the normalised transcript abundance for UPFs (Additional file 1: Table S1 and Additional file 3: Table S3) and CD epitopes and sequence variants of those epitopes. Across all samples (94 cDNA samples, taken from 77 individual plants of 61 accessions), the dominant alpha-gliadin protein fragment was UPF-P1 whereas all others were only present in subsets of the plants analysed (Table 4). To analyse the differential UPF profiles of the wheat samples a hierarchical clustering (Pearson’s correlation, average linkage) was carried out. Based on the normalised transcript abundances and differential presence of the UPFs the samples clustered into ten groups which suggests ten different profiles for expressed alpha-gliadins (alpha-gliadin expression profile 1 to 10) (Figure 5). Due to the concerted presence of UPF-P1 and subsets of lowly abundant UPFs, the correlation among the different UPF profiles was high. The differential expression of several highly abundant (normalised transcript abundance >2) UPF components alone already enabled to distinguish the ten distinct alpha-gliadin expression profiles (Table 4).

**Table 4:** The main unique alpha-gliadin protein fragments in ten different alpha-gliadin profiles

**UPF**	**1**	**2**	**3**	**4**	**5**	**6**	**7**	**8**	**9**	**10**	**DQ2.5-glia-α1 to -α3 fragment**
**P1**	32	25	60	60	62	67	54	31	42	20	PFPQPQLPY**S**QPQPFRPQQPYPQPQPQY
**P2**		7			15			15	36	23	PFPQPQL**S**Y**S**QPQPFRPQQPYPQPQPQY
**P3**			18					6			PFPQPQLPY**SH**PQPFRPQQPYPQPQPQY
**P4**		5									PF**L**QPQLPY**S**QPQPFRPQQPYPQPQPQY
**P5**						15					PFPQPQLPY**SH**PQPFRPQQPYPQPQPQY
**P6**						9					PFPQPQLPY**S**QPQPFRPQQPYPQPQPQY
**P7**		5		8	9				4	15	PFPQPQLPY**S**QPQPFRPQQPYPQPQPQY
**P8**			6								PFPQPQLPY**S**QPQPFRPQQPYPQPQPQY
**P9**							19			3	PFPQPQLPY**S**QPQPFRPQQPYPQPQPQY
**P10**				14							PFPQ**L**QLPY**S**QPQPFRPQQPYPQPQPQY
**P11**					3						PF**-LP**QLPYPQPQPF**P**PQQPYPQPQPQY
**P12**										8	PFPQPQLPY**S**QPQPFRPQQPYPQPQP**R**Y
**P13**	25										PFPQPQLPY**S**QPQPF**Q**PQQPYPQPQPQY
**P14**		7									PF**S**QPQLPY**S**QPQPFRPQQPYPQPQPQY
**P17**				10							PFPQPQLPY**S**QPQPFRPQQPYPQPQPQY
**P18**	20										PFPQPQLPY**S**QPQPFRPQQPYPQPQPQY
**P19**								4		5	PFPQPQLPY**S**QPQPFRPQQPYPQPQP**H**Y
**P20**							7				PFPQPQLPY**L**QPQPFRPQQPYPQPQPQY
**P22**									5	7	PF**S**QPQLPY**L**QPQ**L**FRPQQPYPQPQPQY
**P23**							9				PFP**P**PQLPY**S**QPQPFRPQQPYPQPQPQY
**P25**	7										PFPQPQLPY**S**QPQPFRPQQPYPQPQPQY
**P27**					5						PFPQPQLPY**L**QPQPFRPQQPYPQPQPQY
**P28**								6			PF**L-**PQLPYPQPQPF**P**PQQ**S**YPQPQPQY
**P31**				4							PFPQPQLPY**L**QPQPFRPQQPYPQPQPQY
**P33**								3			PFP**-**PQLPYPQPQ**S**F**P**PQQPYPQ**Q**QPQY
**P35**										4	PFPQPQLPY**S**QPQPFRPQQPYPQPQPQY
**P37**								4			PFPQPQLPY**S**QPQPFRPQQPYPQPQPQY
**P39**								4			PFPQPQLPY**S**QPQPFRPQQPYPQPQPQY
**P42**								3			PFP**-**PQLPYPQ**A**QPFP**T**QQPYPQPQPQY
**P43**		11									PFPQPQLPYPQPQPFRPQQPYPQPQPQY
**P46**								3			PFPQPQLPY**S**QPQPFRPQQPYPQPQPQY
**P49**		9									PFP**P**PQLPY**S**QPQPFRPQQPYPQPQPQY
**P55**								3			PFPQPQLPY**L**QPQPFRPQQPYPQPQPQY
**P62**		7									PFPQPQLPYPQPQPFRPQQPYPQPQPQY
**P77**		4									PFPQPQLPYPQPQPFRPQQPYPQPQPQY
**P82**		3									PFPQPQLPYPQPQLPYPQPQPFRPQQPYPQPQPQY
**Total (%)**	84	83	84	96	94	91	89	82	87	85	

The main UPFs for expression profiles (n = 10) and their average normalised transcript abundances are shown. The main UPF of profiles have an average normalised transcript abundance > 2, in average over all samples with a specific profile, and are expressed in all the plants with that profile. The part of the amino acid sequence of the UPFs that harbours DQ2.5-glia α1 to -α3 is depicted, with P to S substitutions and other substitutions depicted in bold; underlined are canonical DQ2.5-glia a1, -α2 and α3 epitopes. Each expression profile, group 1–10, represents a total of respectively, 33998, 2779, 181184, 37917, 28857, 19863, 33105, 36973, 43252 and 3019 alpha-gliadin transcript 454 sequences. In case of identical amino acid fragments, the different proteins can be distinguished by differences in sequences outside the depicted sequence.

### Reproducibility of the analysis

**Figure 6:**
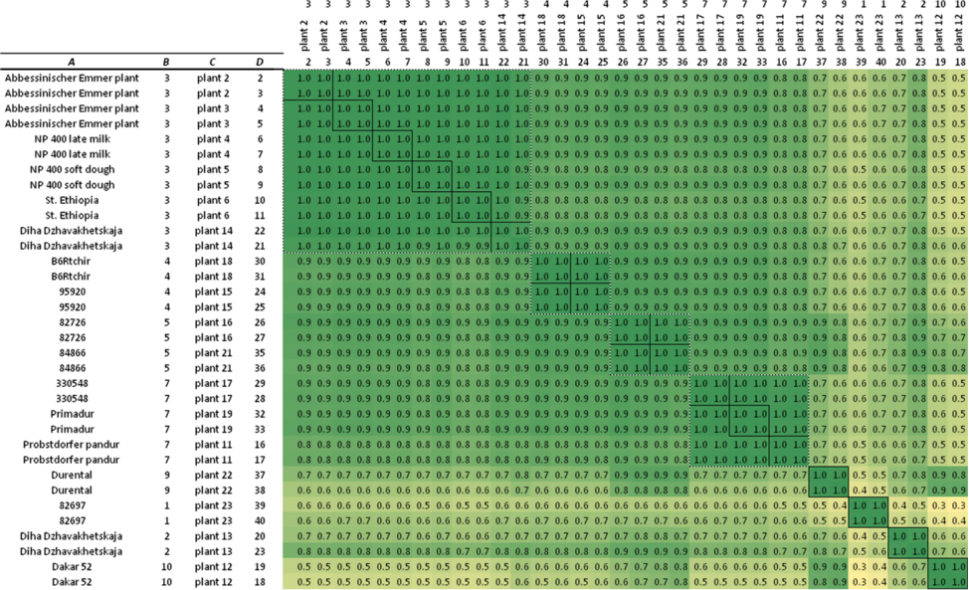
**Correlations matrix of expression profiles.** Comparison of the alpha-gliadin expression profiles found in plants sampled from durum wheat accessions (Pearson’s r). Two cDNA samples were analysed from each plant. **A** = accession name; **B** = expression profile number; **C** = plant number; **D** = cDNA sample number.

To test the reproducibility of the analysis, in 17 cases duplicate cDNA samples from developing seeds of the same plant were analysed. The correlation between samples from the same plants (biological replicates) was high (Pearson’s r =1), indicating a very good reproducibility of the analysis (Figure 6). The breeding line ‘Primadur’ was included in two 454 runs to confirm the technical reproducibility of an alpha-gliadin expression pattern over two 454 runs. The characteristic components UPF-P1, -P9, -P20 and -P23 of alpha-gliadin expression profile 7 (Table 4) were reproducible over the runs (correlation coefficient of expression profiles over two runs, Pearson’s r = 0.99) (Additional file 4: Table S4).

### Sensitivity of the analysis

The 454 sequence analysis pipeline showed to be a sensitive platform to detect individual wheat plants with specific alpha-gliadin expression profiles. Plants that share the same alpha-gliadin expression profile can be recognized by the presence of characteristic UPF components (Table 4) together with a high correlation (Pearson’s r = 0.99) in pairwise comparisons between profiles (Figure 6).

Among plants that share the same alpha-gliadin expression profile, besides the UPFs that are characteristic for the profiles, minor differences in the profile were detectable. For instance, two different plants of accession Primadur (plant 20 and plant 19), both with expression profile 7 (Pearson’s r = 0.99), showed differences in the range of the lower abundant alpha-gliadins components (Additional file 4: Table S4). For the breeding line ‘NP400’ two different stages of seed development were analysed (late milk and soft dough, plant 4 and plant 5, Additional file 1: Table S1). The main components of expression profile 3 were present in both developmental stages and also in this case only several lowly abundant UPFs were differentially present between plants and developmental stages (Additional file 4: Table S4). The same observation was made for differences in the year of cultivation (e.g. CGN07975), indicating the stable expression of sets of highly expressed alpha-gliadin gene variants during endosperm development whereas the presence of several ‘lowly abundant’ alpha-gliadins (normalised transcript abundance ≤ 2) is more variable among plants and under different conditions. Alternatively, at low abundance they are less consistently detected.

**Figure 7:**
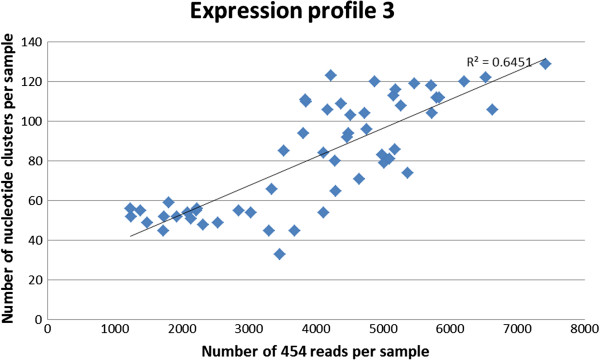
**Sequence depth and number of unique alpha gliadin gene clusters.** The correlation (Pearson’s r) between the sequence read depth of a sample and the number of different unique alpha-gliadin gene clusters detected in plants (n = 49) with expression profile 3.

Differences in sequence depth may influence the number of lowly expressed alpha-gliadins detected. For 49 plants (6 sampled in duplo) with expression profile 3 the number of unique nucleotide clusters increased (range 33 to 129) with the number of analysed 454-reads per sample (Figure 7).

### Heterogeneity within accessions

**Figure 8:**
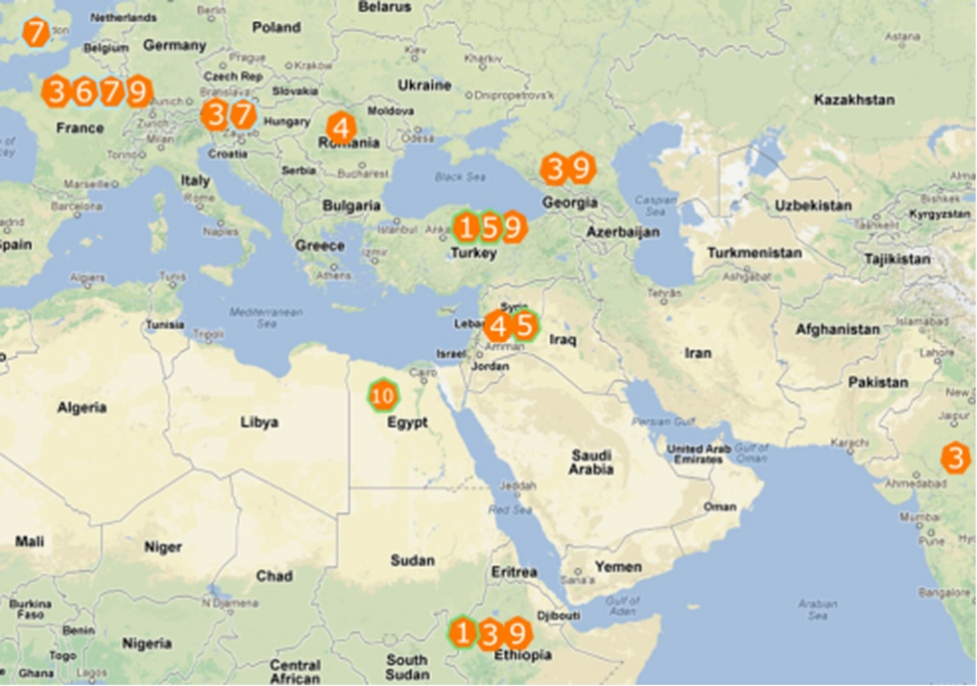
**Geographical distribution of alpha-gliadin expression profiles.** Numbers 1 to 10 are the different alpha-gliadin expression profiles observed in the *T. turgidum* genotypes. Some profiles are only found in Northern regions (6, 7) whereas others are limited to Southern regions (10, 5 and 1).

On the basis of transcript profiles of abundant UPFs (normalised transcript abundance >2) it was possible to distinguish ten alpha-gliadin expression profiles. Most (33 of 37) accessions from CIMMYT showed little variation in alpha-gliadin expression profile, and grouped together in a cluster of 49 plants (55 samples) with expression profile 3 (Figure 5). For landraces more variation within accessions was observed; ‘St. 472 Ethiopia’ (CGN07991), Diha Dzhavakhetskaja (CGN08360) and ‘Dibillik Sinde’ (CGN08006) harboured a mixture of genotypes with different expression profiles. Among the five Dibillik Sinde plants that were analysed two different expression profiles were observed, expression profile 1 and 5. In a mutual comparison of these two alpha-gliadin expression profiles of the five Dibillik Sinde plants, identical expression profiles showed a high correlation (Pearson’s r = 0.9) whereas among different expression profiles a lower correlation was observed (Pearson’s r = 0.6 to 0.8) indicating the sensitivity of the 454 sequence analysis method to distinguish the different alpha-gliadin expression profiles. The material sampled from different regions was diverse, and a unique alpha-gliadin variation (expression profile 10) was found in the Egyptian durum wheat accession ‘Dakar52’. Expression profiles 6 and 7 only occurred in material from Western Europe. Plants with expression profiles 10, 5 and 1 were found in material from the Southern parts of the geographical region (Middle East, Turkey, Ethiopia) (Figure 8). Each of the 10 alpha-gliadin expression profiles included several dominant alpha-gliadin protein variants (UPFs with normalised transcript abundance >2; Table 4) and some of these harboured amino acid changes in the CD epitope region that, according to Mitea et al. [11], may eliminate the potential immunogenicity of the CD epitopes cores.

### CD epitope abundance across plants

**Figure 9:**
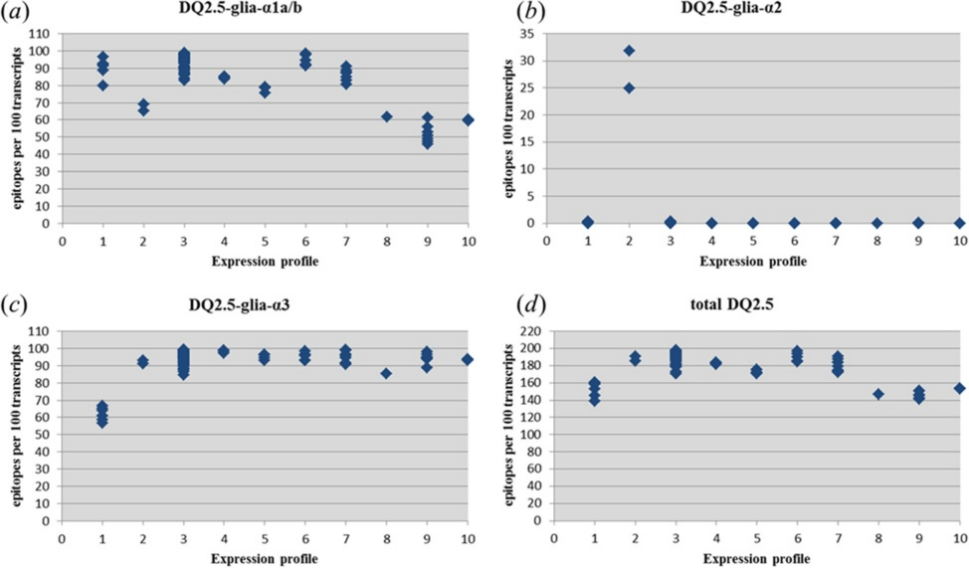
**CD epitope abundance in different alpha-gliadin expression profiles.** Among the 77 plants ten different alpha-gliadin expression profiles are recognized (expression profile 1–10). The number of CD-epitopes per 100 transcripts is shown for each profile (profile 1 to 10) for respectively **(a)** DQ2.5-glia-α1a and DQ2.5-glia-α1b, **(b)** DQ2.5-glia-α2, **(c)** DQ2.5-glia-α3 and **(d)** the total HLADQ2.5 epitopes. Each data point is, the number per 100 transcripts for CD epitopes = ∑171 UPFs (number per UPF for a CD epitope core x normalised transcript abundances per UPF), for a single plant.

The CD epitopes DQ2.5-glia-α1 and DQ2.5-glia-α3 were present in all plants (Figure 9a and 9c, Additional file 1). However, in several plants with expression profile 9 (Figure 9a) the normalised abundance of transcripts coding for DQ2.5-glia-α1 was reduced by half. This was observed in 1509 CM, ‘82715’, ‘Durental’ , with normalised transcript abundances for DQ2.5-glia-α1 of only respectively 47, 49 and 46 to 51. For CD epitope DQ2.5-glia-α3 the encoding transcript abundance in the endosperm was reduced by more than 40% in several accessions with expression profile 1; e.g. in Dibillik Sinde (CGN08006-2B; normalised transcript abundance 59) and ‘CIM-10182’ (normalised transcript abundance 57, Figure 9c). CD epitope DQ2.5-glia-α2 was only found in high numbers in two out of four plants of the landrace Diha Dzhavakhetskaja (CGN08360, expression profile 2, Figure 9b), which were the only plants that expressed UPFs with a clear D genome signature.

Taken together, sequences coding for DQ2.5-restricted CD epitopes were found in alpha-gliadin transcripts of all durum wheat samples with an average of 1.81 ± 0.16 epitopes per alpha-gliadin transcript (Figure 9d). A selection from landrace Dibillik Sinde (CGN08006-2B, profile 1) with 1.39 epitopes per transcript, the elite durum wheat cultivar Durental (expression profile 9) with 1.41-1.45 epitopes per transcript and selection 1509 CM (expression profile 9) with 1.42 epitopes per transcript scored relative low compared to for instance ‘CIM-10139’ (expression profile 3) with 1.98 epitopes per transcript. The lower number of CD epitopes was due to the presence of alpha-gliadin UPFs with a proline (P) to serine (S) substitution on position p8 of both epitopes DQ2.5-glia-α1 and DQ2.5-glia-α2 (in UPF-P2, Table 4) or an arginine (R) to proline (P) or glutamine (Q) substitution on position p2 in DQ2.5-glia-α3 (UPF-P28, UPF-P11 and UPF-P13, Table 3). Both changes lead to peptides that are found to have lost the capacity to trigger an *in vitro* response in HLA-DQ2.5 restricted T cell clones [11].

## Discussion

The gluten fraction of wheat contains proteins that can trigger a T cell reaction in celiac disease (CD) patients, leading to inflammatory responses in the small intestine. Major CD epitopes, DQ2.5-Glia-α1, -α2 and -α3 [5] are found in the first variable domain of wheat alpha-gliadins. To quantify the CD epitopes and their natural variants present in wheat varieties and accessions, and to investigate the possibility to select wheat varieties with a reduced CD immunogenic potential, a high throughput 454 sequence analysis pipeline was developed here to analyse the epitope-containing region in alpha-gliadin genes of tetraploid durum wheat. This region is between 240 bp and 282 bp (80 to 94 amino acids) long, which is in range with the read-length of 454 sequences [37]. The amplicons sequenced ranged from 173 bp to 321 bp, with a median length of 262 bp, before sequence trimming. To exclude the many pseudogenes present in the alpha-gliadin loci the focus of this study was on alpha-gliadin transcripts, and not on genome sequences [21]. In case of alpha-gliadins most pseudogenes have premature stop-codons at specific positions [21]. Some of these genes may be transcribed, and especially when the stop codon occurs near the 3′ end of the gene they may escape the nonsense-decay mechanism [38]. Such transcripts are part of the amplicons, but this is correct as, whenever the premature stop codon occurs downstream of the CD epitopes coding region of the gene, they can be a source of immunogenic peptides.

The sequence analysis pipeline included a number of steps: sequence truncation, repair of 454 sequence mistakes, two rounds of sequence clustering, identification of unique alpha-gliadin transcripts, unique deduced alpha gliadin protein fragments (UPFs), and CD epitope variants, and count of 454-reads per variant. SNPs and InDels are the most abundant forms of DNA sequence variation in common wheat and its relatives [39], which was confirmed in our alpha-gliadins. The pipeline thus enabled the use of 44% of the 963,666 transcript reads, which coded for an overall number of 171 unique alpha-gliadin protein fragments (UPFs; 18–84 per plant).

Based on conserved amino acid stretches that are indicative for gliadins from the homoeologous loci *Gli-2A* and *Gli-2B*[11,36] expression from the *Gli-2A* locus was estimated to be much higher (normalised transcript abundance of 76–99 per plant) compared to expression from *Gli-2B* ( normalised transcript abundance of 1–20 per plant). Unequal transcription from homoeologous alpha-gliadin loci in wheat has been shown in other studies (e.g. [36]). Also, Kawaura et al. [40] observed that *Gli-2B* transcripts were underrepresented compared to their homoeologs from the A and D genomes in a set of expressed sequence tags from dbEST. Two alpha-gliadin protein fragments with a combined D and B genome signature that were observed in a couple of breeding lines of durum wheat most likely represent D-like alpha-gliadin genes expressed from the B genome. The presence of such alpha-gliadins with an intermixed genomic signature probably reflects their common ancestry. In gamma-gliadins sequences from different genomes are even harder to distinguish as separate groups [41].

### Alpha-gliadin gene expression

The alpha-gliadin promoter is active in the wheat endosperm from 11 days after anthesis until maturity, which is about 4 weeks after anthesis [42]. Kawaura et al. [43] observed that nine of twelve intact alpha-gliadin genes of bread wheat were expressed in distinct patterns during endosperm development, whereas three were not expressed. We have harvested inflorescence spikes at 21 days after anthesis of the first flower, from which seeds from the milk to soft dough developmental stages were selected. The profiles of expressed alpha-gliadins that are shown in the present study were reproducible (among duplicate cDNAs from the same plant, among different plants, over different runs) and stable and the major components (UPFs with a normalised transcript abundance >2) of the profiles were not related to differences in developmental stage or environmental conditions (year of harvest, field or greenhouse conditions).

In comparisons among plants sharing the same expression profile the minor components (UPFs with a normalised transcript abundance ≤ 2) of the expression profiles were more prone to variation, which may be due to differences in sequence depth but also environmental differences and /or differences in the genetic background of plants may play a role.

However, it cannot be excluded that some early or late expressed alpha-gliadin variants may have been missed or are underrepresented but, it is unlikely that many genes are missing because of selective amplification, as the reverse primer used here covers all variants that are present in a set of 3,000 expressed alpha-gliadin sequences from 11 bread wheat cultivars and various tissues and treatments [11] and the forward primer covers all but two variants. Furthermore, all alpha-gliadin ESTs of cultivar ‘Butte 86’ as assembled by Altenbach et al. [44] contained the forward and reverse primer sequences used here.

Several lines of evidence support the occurrence of different expression patterns among alpha-gliadin genes. First, differences among groups of alpha-gliadin ESTs were observed between the genomes [40] and in developing endosperm [43] in terms of numbers of reads per contig. Secondly, using pyrosequencing differences in expression among alpha-gliadins were shown in tetraploids, and some of them had differences in CD epitopes as well [36], although the power of resolution of targeted 454 sequencing as used in the present study is much higher.

Based on the genetic variation in the part of the alpha-gliadin genes analysed here, as many as 24 to 129 different alpha-gliadin genes are expressed in a single tetraploid durum wheat plant from the two combined homoeologous *Gli-2* loci. This number will be approximately doubled when the variation in the signal peptide sequence would be taken into account (results not shown), and even higher when the variation in the repetitive domains towards the 3′-end of the genes, which we did not sequence here, is considered.

### Alpha-gliadin protein profiles

Reproducible and stable gliadin protein profiles are applied as markers in wheat breeding and have been used to study crop genetic diversity in a global collection of durum wheat [45]. Blocks of protein bands in electrophoretic profiles of gliadins inherit as linked groups and display a stable co-dominant inheritance, indicating that they are encoded by alpha-gliadin genes from one *Gli-2* locus. The patterns of blocks are described in detail in a catalogue of alleles [46-48]. Consistent with this, also some of the 171 alpha-gliadin protein fragments (UPFs) showed distinct and reproducible expression profiles among the durum wheat plants and accordingly the protocol presented may be useful to detect genetic variation among wheat varieties. The material from CIMMYT showed low genetic diversity and nearly all of these accessions displayed the alpha-gliadin expression profile 3. Melnikova et al. [45] also observed high similarity in gliadin protein profiles in material from breeding centres, which they considered to be the result of strong selection for plant homogeneity for breeding traits. Some of the alpha-gliadin expression profiles observed by them were exclusively found in material from Northern regions (Russian and Ukrainian accessions) whereas others were only found in material from the South (Mediterranean region, the Middle East, and Trans Caucasia) [45]. Similarly, in the material studied here distinct Southern and Northern alpha-gliadin expression profiles are observed using profiling of an alpha-gliadin RNA-amplicon by 454 sequencing. Unique alpha-gliadin transcript variation was observed in the Egyptian durum wheat accession Dakar 52 (Figure 6).

### Differences in CD epitope composition among durum wheat accessions

A wide variation in both gluten composition and T cell immunological activation was found among tetraploid farro wheats (*Triticum turgidum* ssp. dicoccum) by Vincentini et al. [14], some of these ‘dicoccum’ landraces possibly being low in CD-immunogenic gluten proteins. Here, mainly accessions of *Triticum turgidum* spp. durum were analysed and although some variation in CD epitope content was found, none of the accessions was completely devoid of CD epitopes, as transcripts (454-reads) with the major HLADQ2.5 restricted CD epitopes were present in all samples, albeit at different rates. In accession 1509 CM and the elite cultivar Durental, both having alpha-gliadin expression profile 9, the number of transcripts coding for CD epitope DQ2.5-glia-α1, as determined by normalised transcript abundance, was halved compared to samples with other alpha-gliadin expression profiles. Another accession, a selection from landrace Dibillik Sinde (CGN08006-2B) with expression profile 1, scored low for CD epitope DQ2.5-glia-α3. The reduction in CD epitopes in durum wheat accessions with expression profile 9 is due to the high expression rate of alpha-gliadins with a proline (P) to serine (S) substitution on position p8 in DQ2.5-glia-α1 and DQ2.5-glia-α2, which creates epitope variants that are not capable to trigger an in vitro response in HLA-DQ2.5 restricted T cell clones that are specific for the respective CD epitopes [11]. Using an immunoblotting procedure with monoclonal antibodies against DQ2.5-glia-α1 (mAb-α9) and DQ2.5-glia-α3 (mAb-α20) Van den Broeck et al. [35] also selected several genotypes that are apparently low in both CD epitopes and in line with the results from the RNA-amplicon profiling shown here, a protein extract of a plant of landrace Dibillik Sinde (CGN08006) was selected by these authors as having the lowest affinity for binding with the epitope-specific antibody. Other accessions showed, however, no clear correlation between the two studies. Probably this is due to the limitations of antibodies, which have a shorter minimal recognition site (7-mer rather than the 9-mer T cell epitope) and may not be able to detect all amino acid substitutions. On the other hand transcripts undergo translation before they end up in the protein bodies. In that respect integration of transcriptomic and proteomic data will provide the ultimate tool for determining the CD epitope load in individual wheat plants.

## Conclusions

The dedicated 454 RNA-amplicon sequencing pipeline for alpha-gliadin transcripts can be used as a tool to detect genetic diversity in wheat alpha-gliadins. Using this tool, wheat germplasm can be screened for plants that are potentially less CD-immunogenic. The sequence data obtained in the process are providing a database for further proteomics analysis of the selected plants, regarding the immunogenic potential of the final gluten composition in mature grains. A few plants showed lower normalised transcript abundances for specific CD epitopes, but the fact that major CD epitopes were found to be present in most alpha-gliadin genes and in all accessions tested, indicates that among the durum wheat plants tested no genotype has been found that is safe for CD patients. Moreover, it seems unlikely that conventional selection and breeding within this tetraploid germplasm will lead to the development of varieties that are safe to individuals with CD. For that reason we are currently screening commercial *T. monococcum* spp. (A genome) varieties and accessions. As an alternative, the 454 RNA-amplicon sequencing strategy will be useful to analyse the CD epitope profiles in wheat lines with induced mutations, such as panels of radiation hybrids [49] and deletion lines [50], as potential starting material for breeding of CD-safe wheat.

## Methods

### Plant material

Alpha-gliadin transcript sequences were amplified from developing seeds (cDNA) of 77 plants from 61 different *T. turgidum* accessions. (Additional file 1: Table S1).

The panel of 61 accessions included seven *T. turgidum* accessions obtained from the Centre for Genetic Resources (CGN, Wageningen, The Netherlands) grown under field conditions in the spring and summer (year 2005, 2007) in sandy soils fertilized with Tripelsuperfosfate (45% P2O5) 108.97 kg/ha, Kali60 (60% K2O) 108.97 kg/ha, Kalkammonsalpeter (27% N;NH4NO3 + 6% CaCO3) 275 kg/ha. Furthermore, the panel included 16 accessions from the core collection of tetraploid wheat obtained from the Institut National de la Recherche Agronomique (INRA), Montpellier, France [51] and 38 lines obtained from Limagrain Nederland BV, Lelystad, The Netherlands. The latter two groups were grown in a climatised greenhouse as in [50]*.*

To obtain biological replicates, duplicate cDNA samples were taken from 17 plants (4 to 5 seeds per sample from the same plant and the same spike), giving a total of 94 cDNA samples (77 + 17). The 17 plants that were sampled twice are: plant 2 (CGN07975), plant 3 (CGN07975), plant 4 (CGN06560), plant 5 (CGN06560), plant 6 (CGN07991), plant 11 (CGN08262), plant 12 (CGN16072), plant 13 (CGN08360), plant 14 (CGN08360), plant 15 (INRA328) plant 16 (INRA351), plant 17 (INRA330548), plant 18 (INRA581), plant 19 (INRA395), plant 21 (INRA302), plant 22 (INRA437) and plant 23 (INRA344). The elite cultivar Durental was included in two of the three 454 runs.

On the basis of transcript profiles of abundant UPFs (normalised transcript abundance >2) it was possible to distinguish 10 alpha-gliadin expression profiles. To test heterogeneity for expression profiles within accessions, for eight different accessions several plants were analysed. These accession were: Abbessinischer Emmer (CGN07975, 3 plants analysed), NP 400 (CGN06560, 2 plants), St. 472 Ethiopia (CGN07991, 2 plants), Probstdorfer pandur (CGN08262, 2 plants), Diha Dzhavakhetskaja (CGN08360, 2 plants), Dibillik Sinde (CGN08006, 5 plants), Primadur (2 plants) and Neodur91 (4 plants). The composition of genebank accessions may be genetically heterogeneous because of the goal to preserve genetic variation and CGN08006 and CGN08360 were already known to be mixtures of different genotypes [34]. CGN8360 was confirmed in flow cytometric ploidy level determination to be mixed with hexaploid genotypes that are phenotypically identical to the tetraploids [35]. Breeding material from INRA and Limagrain was expected to be genetically homogeneous within accessions [35]. Details of plant material used are given in Additional file 1: Table S1. The geographic origin of the accessions is included to be able to determine possible geography-related genomic difference in CD epitope quantity and quality.

### RNA extraction, purification and cDNA synthesis

Developing seeds were harvested at 21 days after anthesis of the first flowers (ripening stages of the seeds in the inflorescence spikes ranged from milk to soft dough). The mRNA was extracted from a mixture of 4 to 5 seeds (100 mg maximum) from a single plant by grinding in 750 μl of Trizol followed by incubation at room temperature for 5 minutes. After extraction with 150 μl of chloroform, 200 μl of the supernatant was transferred to a clean 1.5 ml tube. Subsequently, the RNA was purified using the RNeasy MiniKit (Qiagen GmbH, Hilden, Germany) and eluted in 30 μl RNase free water. One microliter of the elutate was used to check RNA quality on a spectrophotometer (NanoDrop ND1000, NanoDrop products, Wilmington, Delaware, USA) and three microliters, stained with GelRed, were used for visual inspection of the RNA quality on agarose gel, 1% (w/v). The final concentration of RNA as measured by spectrophotometer ranged from 25 ng/μl to over 1000 ng/μl.

### DNAse I treatment and cDNA synthesis

DNA treatment (TURBO DNA-free (Ambion, Austin, Texas, USA) and cDNA sythesis (iScript cDNA synthesis kit, BioRad Laboratories Inc., California, USA) were performed according to vendor protocols.

### PCR amplification

The alpha-gliadin amplicons were prepared in two steps. The first amplification was done using gene specific primers, Alpha1Fdeg454 (5′-atgaaracmtttcycatc-3′; for the MKTF[LP]I- motif, Figure 1) and AlphaR454 (5′-ctgctgctgtgaaattrgwt-3′; for the PISQQQ-motif, Figure 1). For each of the 94 samples this amplification event was replicated on three different PCR machines (3 × 94 PCRs) to minimise amplification bias while increasing the reliability and validity of the amplification results. Amplification was performed in 20 μl reaction containing 4 μl Phusion buffer, 0.8 μl dNTP (5 mM), 0.25 μl Adaptor primer (10 pmol/μl), 0.25 μl Specific primer (10 pmol/μl), 12.6 μl MQ, 0.1 μl Phusion High-Fidelity DNA polymerase (2 Units/μl, Finnzymes-Thermo Scientific, Massachusetts, USA) and 2 μl cDNA. The PCR cycling conditions were 98°C for 30 sec followed by 30 cycles of {98°C for 5 sec, 50°C for 10 sec and 72°C for 30 sec}, 5 min at 72°C. Next, for each sample, the products of the three amplifications were pooled together and 10 μl of this mixture was used as a template for a second PCR amplification event, applying fusion primers (Additional file 5: Table S5) that included the sequences needed for 454 sequencing, a gene specific part and a 10 bp ID sequence in a 30 μl reaction volume containing 4 μl Phusion buffer, 0.8 μl dNTP (5 mM), 0.5 μl 454-Adaptor primer (10 pmol/μl), 0.5 μl 454-Specific primer (10 pmol/μl), 14.1 μl MQ, 0.1 μl Phusion DNA polymerase, using otherwise the same PCR conditions and again on three different PCR machines (3 × 94 PCRs). After this second PCR step the three amplifications of a sample were pooled resulting in 94 samples that were sequenced in three 454 sequencing runs: two quarter 454 runs of respectively 23 and 24 samples (run 1 id. F4SOYM002 and run 2 id. GL4NQHJ02) and a half 454 run of 47 samples (run 3 id. G6WZP5402). The samples for each run were equimolarly pooled (super-pools of respectively 23, 24 and 47 samples) and subsequently each of the three super-pools was size fractioned using a 1% (w/v) agarose gel. The fragments of 350–400 bp were cut from the gel, purified (gel extraction kit, Qiagen GmbH, Hilden, Germany) and sent for sequencing (Roche/454 sequencing, dr. Elio GWM Schijlen, Plant Research International, Wageningen, The Netherlands).

Each sample for 454 sequencing contained alpha-gliadin transcripts derived from developing seeds (cDNA) of a single plant labelled with a unique10 bp ID sequence. In the first run several samples that contrasted in developmental stage were analysed separately; e.g. for CGN06560 plant 4, late milk stage and plant 5, soft dough stage. In the other two runs 4 to 5 seeds were sampled from a single spike whereby developmental stages were mixed. In total, the developing seeds of 77 plants were analysed of which 17 in duplicate (see section Plant material) to test the reproducibility of the analysis, giving a total of 94 samples. The statistics for each run are given in Table 1.

### 454 sequence analysis

Roche/454 amplicon sequencing resulted in over 900,000 alpha-gliadin transcript sequences (454-reads; minimum length 173 bp, median length 262 bp, maximum length 321 bp, before sequence trimming). Pre-processing of the transcripts, using custom PERL scripts, involved renaming the sequences after the barcode-ID, allowing a mismatch of 1 bp, trimming of the sequences to the repetitive domain of the alpha-gliadins (Figure 1, blue underlined); removal of the barcode, the forward alpha-gliadin primer sequence and the signal peptide, trimming of the reverse alpha-gliadin primers; and correction of typical homopolymer 454-read errors.

The resulting 628,845 transcript sequences, after trimming 240 bp to 281 bp in size and harbouring the major CD epitopes, were then clustered using USEARCH V4.0. First, sequences were sorted by decreasing abundance since the most abundant sequence is likely to be a correct sequence, while less common sequences may include artefacts due to sequencing errors or PCR artefacts. This sorting by decreasing abundance was accomplished by clustering the sequences at 100% homology (every gap and every mismatch counted as a difference) and counting the number of sequences per cluster. Next, the clusters were sorted by decreasing cluster size and subsequently the ‘cluster representative sequences’ of clusters with more than 19 sequences per sequence run were clustered at 99.5% homology. After this clustering step, only clusters with more than 20 sequences across all 94 samples passed (total 422,301 sequences). The output of the pipeline consisted of the consensus cDNA sequences of these clusters (a total of 1,421 unique consensus DNA sequences), the deduced amino acid sequences (215 unique deduced protein fragments, abbreviated as UPFs), the number of 454-reads per cluster per sample, and the number of CD epitopes per UPF. An overview of the 454 sequence analysis pipeline is shown in Figure 2.

### Data analysis

The 454-read counts were normalised by calculating (‘454-read count per UPF’ / ‘total 454-read count of a sample’) × 100 and named ‘normalised transcript abundance’.

Only UPFs that were expressed at a threshold of a normalised transcript abundance of at least 0.1 and had a coverage (454-read count) of more than 5 sequences in at least one of the 94 samples were taken into account. Remaining for analysis were a total number of 420,947 sequences (44% of the raw 454-reads; range 1,228 to 10,211 per sample, average 4,478 per sample), organized into 304 unique sequence clusters (range 24–129 clusters per sample, average 68 per sample) (Additional file 2) that coded for 171 UPFs) representing the variants of the first variable domain of alpha-gliadins that were expressed in the endosperm (range 18–84 UPFs per sample, average 50 per sample; length 80 to 94 amino acids).

The final library of 171 UPFs was screened for variation in the amino acid sequences of the CD epitopes DQ2.5-Glia-α1, DQ2.5-Glia-α2 and DQ2.5-Glia-α3 in their non-deamidated forms (respectively PFPQPQLPY, PQPQLPYPQ and FRPQQPYPQ). Subsequently, the normalised transcript abundances for UPFs and CD epitopes and their variants were calculated.

### Statistics

Hierarchical cluster analysis (Pearson’s r, average linkage, distance threshold 2.075) of samples on basis of the normalised transcript abundances of UPFs was performed using MultiExperiment Viewer (MeV) software [52]. Other calculations were performed in Excel.

### CD immunogenic potential

An estimate for the CD immunogenic potential was calculated by scoring the number of canonical HLA-DQ2.5 CD epitope sequences, listed in Table 1 (in bold underlined), per UPF and calculating the CD epitope frequency per transcript (454-read) and per sample. The epitope frequency of a sample was calculated as ‘CD immunogenic potential’ = ∑ 171 UPFs (number of canonical DQ2.5 CD epitopes per UPF x normalised transcript abundances per UPF)/100. In addition, all different sequence variants of the CD epitopes were scored. The number per transcript for a single CD epitope was calculated as ∑171 UPFs (number of that CD epitope core per UPF x normalised transcript abundances per UPF)/100.

### Sequence alignment and motif search

Sequence alignment and Neighbor-Joining analysis of deduced unique alpha-gliadin protein fragments (UPFs) was performed using MEGA version 5 [53]. The sequences were assigned to a sub-genome of wheat based on the presence of distinct sub-genome specific amino acid motifs [11,21] (Table 2).

## Competing interest

The authors declare that they have no competing interests.

## Authors’ contributions

EMJS, DE and MJMS participated in the design of the study. SVG carried out 454 sequencing; DE carried out 454 sequencing and designed the sequence analysis pipeline; EMJS performed sequence analysis; EMJS, DE, IMM, LJWJG, MJMS drafted the manuscript. All authors read and approved the final manuscript.

## Supplementary Material

Additional file 1: Table S1 Details of wheat accessions used, samples for 454 sequencing, and summary of results of sequence analysis. Click here for file

Additional file 2 **Unique alpha-gliadin nucleotide fragments.** Unique alpha-gliadin nucleotide fragments expressed in the durum wheat varieties analysed. Click here for file

Additional file 3: Table S3 Normalised expression values. Click here for file

Additional file 4: Table S4 Reproducibility. Normalised transcript abundances for wheat accession Primadur over two 454 runs (plant 19 in duplo in run 2 and plant 20 in run 3) and for NP400 in a comparison of seed developmental stage late milk (Lm) (plant 4) and the soft dough (Sd) staged (plant 5). In green: major and characteristic components of expression profile 7 and profile 3. In grey with black letters: differential abundance among samples of the same accession. In grey with white letters: conserved abundance among samples of the same accession. Click here for file

Additional file 5: Table S5 Fusion Primers for 454 sequencing of alpha-gliadins. Fusion primers contain sequences needed for 454 sequencing and a gene-specific part (underlined). A 10 bp ID sequence (in bold) that enabled the identification of sequences from a particular sample was present in the forward primers. The products of three amplifications were pooled together and used as a template for a second PCR amplification event using these fusion primers. Click here for file
