# Supplementary material for: Quantitative and qualitative differences in celiac disease epitopes among durum wheat varieties identified through deep RNA-amplicon sequencing
Source: BMC Genomics. 2013 Dec 19;14:905. doi: 10.1186/1471-2164-14-905 (PMC3890609; doi:10.1186/1471-2164-14-905)
Supplement: Additional file 4: Table S4 — Reproducibility. Normalised transcript abundances for wheat accession Primadur over two 454 runs (plant 19 in duplo in run 2 and plant 20 in run 3) and for NP400 in a comparison of seed developmental stage late milk (Lm) (plant 4) and the soft dough (Sd) staged (plant 5). In green: major and characteristic components of expression profile 7 and profile 3. In grey with black letters: differential abundance among samples of the same accession. In grey with white letters: conserved abundance among samples of the same accession. [file 1471-2164-14-905-S4.docx]

| **Primadur** | | | | **NP400** | | | | |
| --- | --- | --- | --- | --- | --- | --- | --- | --- |
| **Expression profile 7** | | | | **Expression profile 3** | | | | |
|  | **run 2** | **run 2** | **run 3** |  | **Lm** | **Lm** | **Sd** | **Sd** |
| **Sample** | **33** | **32** | **34** | **Sample** | **6** | **7** | **8** | **9** |
| **UPF** | **Plant 19** | **plant 19** | **Plant 20** | **UPF** | **Plant 4** | **Plant 4** | **Plant 5** | **Plant 5** |
| **P1** | 61.63 | 57.94 | 58.90 | **P1** | 53.31 | 55.68 | 51.39 | 55.19 |
| **P9** | 16.44 | 17.57 | 20.84 | **P3** | 19.28 | 18.67 | 24.08 | 20.51 |
| **P23** | 7.63 | 9.47 | 9.00 | **P2** | 0.00 | 0.24 | 0.00 | 0.00 |
| **P20** | 6.55 | 5.81 | 3.79 | **P8** | 6.41 | 8.41 | 7.03 | 6.15 |
| **P28** | 0.42 | 0.43 | 2.33 | **P9** | 0.42 | 0.43 | 0.56 | 0.91 |
| **P50** | 0.40 | 0.21 | 0.75 | **P7** | 0.00 | 0.16 | 0.00 | 0.00 |
| **P44** | 0.22 | 0.50 | 0.66 | **P6** | 1.25 | 0.28 | 0.67 | 1.10 |
| **P3** | 0.00 | 0.00 | 0.57 | **P28** | 0.89 | 4.18 | 1.02 | 1.20 |
| **P33** | 0.06 | 0.21 | 0.49 | **P5** | 0.16 | 0.00 | 0.04 | 0.00 |
| **P42** | 0.00 | 0.00 | 0.40 | **P11** | 0.52 | 0.87 | 0.46 | 0.58 |
| **P26** | 0.11 | 0.16 | 0.37 | **P16a** | 1.56 | 0.47 | 2.53 | 2.02 |
| **N13** | 0.00 | 0.00 | 0.26 | **P20** | 0.10 | 0.00 | 0.00 | 0.00 |
| **N91** | 0.00 | 0.00 | 0.23 | **P33** | 1.30 | 2.05 | 1.30 | 0.82 |
| **P2** | 0.16 | 0.18 | 0.20 | **P37** | 2.50 | 0.24 | 0.53 | 0.72 |
| **N176** | 0.01 | 0.01 | 0.20 | **N6** | 0.57 | 0.43 | 0.56 | 0.62 |
| **N6** | 0.00 | 0.00 | 0.17 | **P39** | 1.09 | 0.16 | 0.60 | 0.34 |
| **N127** | 0.00 | 0.00 | 0.11 | **P46** | 0.26 | 0.12 | 0.00 | 0.00 |
| **N17** | 0.00 | 0.00 | 0.09 | **P55** | 0.36 | 0.04 | 0.35 | 0.24 |
| **N239** | 0.00 | 0.00 | 0.09 | **P30** | 2.03 | 3.91 | 2.04 | 2.02 |
| **P35** | 0.00 | 0.00 | 0.06 | **P32** | 0.42 | 0.47 | 0.04 | 0.29 |
| **N87** | 0.16 | 0.36 | 0.06 | **P4** | 0.00 | 0.04 | 0.00 | 0.00 |
| **N144** | 0.00 | 0.00 | 0.06 | **P19** | 0.00 | 0.04 | 0.00 | 0.05 |
| **P17** | 0.26 | 0.24 | 0.03 | **P44** | 0.21 | 0.08 | 0.56 | 0.19 |
| **P4** | 0.12 | 0.11 | 0.03 | **P26** | 0.36 | 0.16 | 0.32 | 0.48 |
| **N8** | 0.00 | 0.00 | 0.03 | **N17** | 0.31 | 0.04 | 0.25 | 0.67 |
| **N213** | 0.00 | 0.00 | 0.03 | **P35** | 0.42 | 0.20 | 0.39 | 0.48 |
| **N174** | 0.00 | 0.00 | 0.03 | **N87** | 0.00 | 0.00 | 0.25 | 0.05 |
| **N65** | 0.02 | 0.03 | 0.03 | **P12** | 0.05 | 0.04 | 0.07 | 0.00 |
| **N202** | 0.00 | 0.00 | 0.03 | **P41** | 0.89 | 0.20 | 0.28 | 0.53 |
| **N242** | 0.00 | 0.00 | 0.03 | **N4** | 0.05 | 0.00 | 0.25 | 0.62 |
| **N246** | 0.00 | 0.00 | 0.03 | **N15** | 0.21 | 0.04 | 0.18 | 0.14 |
| **N196** | 0.00 | 0.00 | 0.03 | **N176** | 0.00 | 0.08 | 0.14 | 0.10 |
| **N133** | 0.00 | 0.00 | 0.03 | **P36** | 1.15 | 0.59 | 0.81 | 0.43 |
| **N203** | 0.00 | 0.00 | 0.03 | **N8** | 0.00 | 0.04 | 0.25 | 0.10 |
| **N100** | 0.00 | 0.00 | 0.03 | **P15** | 1.25 | 0.83 | 0.63 | 0.62 |
| **P11** | 2.20 | 2.68 | 0.00 | **P47** | 0.26 | 0.00 | 0.63 | 0.43 |
| **P32** | 1.03 | 1.05 | 0.00 | **N11** | 0.16 | 0.00 | 0.21 | 0.14 |
| **N191** | 0.45 | 0.31 | 0.00 | **N144** | 0.31 | 0.08 | 0.11 | 0.14 |
| **P54** | 0.36 | 0.51 | 0.00 | **P38** | 0.16 | 0.00 | 0.04 | 0.14 |
| **N192** | 0.31 | 0.13 | 0.00 | **P14** | 0.00 | 0.00 | 0.00 | 0.05 |
| **P39** | 0.22 | 0.09 | 0.00 | **P49** | 0.00 | 0.12 | 0.00 | 0.00 |
| **N108** | 0.21 | 0.25 | 0.00 | **N20** | 0.16 | 0.00 | 0.14 | 0.00 |
| **P7** | 0.12 | 0.05 | 0.00 | **N174** | 0.16 | 0.04 | 0.18 | 0.14 |
| **N102** | 0.21 | 0.10 | 0.00 | **N161** | 0.10 | 0.12 | 0.07 | 0.19 |
| **P29** | 0.15 | 0.43 | 0.00 | **P51** | 0.21 | 0.24 | 0.18 | 0.29 |
| **N186** | 0.09 | 0.18 | 0.00 | **N82** | 0.00 | 0.00 | 0.04 | 0.00 |
| **N82** | 0.11 | 0.20 | 0.00 | **N118** | 0.16 | 0.04 | 0.18 | 0.29 |
| **N46** | 0.07 | 0.15 | 0.00 | **N18** | 0.16 | 0.04 | 0.00 | 0.00 |
| **P45** | 0.04 | 0.13 | 0.00 | **P111** | 0.16 | 0.00 | 0.00 | 0.14 |
| **N178** | 0.04 | 0.10 | 0.00 | **N119** | 0.26 | 0.08 | 0.21 | 0.38 |
| **N18** | 0.04 | 0.14 | 0.00 | **N10** | 0.16 | 0.04 | 0.21 | 0.29 |
| **N184** | 0.02 | 0.15 | 0.00 | **N19** | 0.26 | 0.04 | 0.28 | 0.19 |
| **N104** | 0.04 | 0.06 | 0.00 |  |  |  |  |  |
| **P5** | 0.01 | 0.00 | 0.00 |  |  |  |  |  |
| **P18** | 0.01 | 0.00 | 0.00 |  |  |  |  |  |
| **N103** | 0.01 | 0.00 | 0.00 |  |  |  |  |  |
| **N101** | 0.02 | 0.00 | 0.00 |  |  |  |  |  |
| **P13** | 0.00 | 0.03 | 0.00 |  |  |  |  |  |
| **P6** | 0.00 | 0.03 | 0.00 |  |  |  |  |  |
| **Total%** | **100** | **100** | **100** | **Total%** | **100** | **100** | **100** | **100** |
| **Ntotal** | **8043** | **7984** | **3479** | **Ntotal** | **1919** | **2534** | **2845** | **2082** |
| **N UPF** | **37** | **37** | **35** | **N UPF** | **42** | **41** | **42** | **41** |

**Additional file 4: Table S4**

Normalized transcript abundances for wheat accession Primadur over two 454 runs (plant 19 in duplo in run 2 and plant 20 in run 3) and for NP400 in a comparison of seed developmental stage late milk (Lm) (plant 4) and the soft dough (Sd) staged (plant 5). In green: major and characteristic components of respectively expression profile 7 and profile 3. In grey with black letters: differential abundant among samples of the same accession. In grey with white letters: conserved abundant among samples of the same accession.

Expression profile 7, R^2^

| Primadur | | | |
| --- | --- | --- | --- |
|  | plant 19 (sample 33) | plant 19 (sample 32 | plant 20 (sample 34) |
| plant 19(sample 33) | x | 0.997 | 0.988 |
| plant 19(sample 32) | 0.997 | x | 0.993 |
| plant 20 (sample 34) | 0.988 | 0.993 | x |
