# Supplementary material for: Quantitative and qualitative differences in celiac disease epitopes among durum wheat varieties identified through deep RNA-amplicon sequencing
Source: BMC Genomics. 2013 Dec 19;14:905. doi: 10.1186/1471-2164-14-905 (PMC3890609; doi:10.1186/1471-2164-14-905)
Supplement: Additional file 5: Table S5 — Fusion Primers for 454 sequencing of alpha-gliadins. Fusion primers contain sequences needed for 454 sequencing and a gene-specific part (underlined). A 10 bp ID sequence (in bold) that enabled the identification of sequences from a particular sample was present in the forward primers. The products of three amplifications were pooled together and used as a template for a second PCR amplification event using these fusion primers. [file 1471-2164-14-905-S5.docx]

| Alphagli454F01 | 5’-CCATCTCATCCCTGCGTGTCTCCGACTCAG**ACGAGTGCGT**ATGAARACMTTTCYCATC-3’ |
| --- | --- |
| Alphagli454F02 | 5’-CCATCTCATCCCTGCGTGTCTCCGACTCAG**ACGCTCGACA**ATGAARACMTTTCYCATC-3’ |
| Alphagli454F03 | 5’-CCATCTCATCCCTGCGTGTCTCCGACTCAG**AGACGCACTC**ATGAARACMTTTCYCATC-3’ |
| Alphagli454F04 | 5’-CCATCTCATCCCTGCGTGTCTCCGACTCAG**AGCACTGTAG**ATGAARACMTTTCYCATC-3’ |
| Alphagli454F05 | 5’-CCATCTCATCCCTGCGTGTCTCCGACTCAG**ATCAGACACG**ATGAARACMTTTCYCATC-3’ |
| Alphagli454F06 | 5’-CCATCTCATCCCTGCGTGTCTCCGACTCAG**ATATCGCGAG**ATGAARACMTTTCYCATC-3’ |
| Alphagli454F07 | 5’-CCATCTCATCCCTGCGTGTCTCCGACTCAG**CGTGTCTCTA**ATGAARACMTTTCYCATC-3’ |
| Alphagli454F08 | 5’-CCATCTCATCCCTGCGTGTCTCCGACTCAG**CTCGCGTGTC**ATGAARACMTTTCYCATC-3’ |
| Alphagli454F09 | 5’-CCATCTCATCCCTGCGTGTCTCCGACTCAG**TCTCTATGCG**ATGAARACMTTTCYCATC-3’ |
| Alphagli454F10 | 5’-CCATCTCATCCCTGCGTGTCTCCGACTCAG**TGATACGTCT**ATGAARACMTTTCYCATC-3’ |
| Alphagli454F11 | 5’-CCATCTCATCCCTGCGTGTCTCCGACTCAG**CATAGTAGTG**ATGAARACMTTTCYCATC-3’ |
| Alphagli454F12 | 5’-CCATCTCATCCCTGCGTGTCTCCGACTCAG**CGAGAGATAC**ATGAARACMTTTCYCATC-3’ |
| Alphagli454F13 | 5’-CCATCTCATCCCTGCGTGTCTCCGACTCAG**ATACGACGTA**ATGAARACMTTTCYCATC-3’ |
| Alphagli454F14 | 5’-CCATCTCATCCCTGCGTGTCTCCGACTCAG**TCACGTACTA**ATGAARACMTTTCYCATC-3’ |
| Alphagli454F15 | 5’-CCATCTCATCCCTGCGTGTCTCCGACTCAG**CGTCTAGTAC**ATGAARACMTTTCYCATC-3’ |
| Alphagli454F16 | 5’-CCATCTCATCCCTGCGTGTCTCCGACTCAG**TCTACGTAGC**ATGAARACMTTTCYCATC-3’ |
| Alphagli454F17 | 5’-CCATCTCATCCCTGCGTGTCTCCGACTCAG**TGTACTACTC**ATGAARACMTTTCYCATC-3’ |
| Alphagli454F18 | 5’-CCATCTCATCCCTGCGTGTCTCCGACTCAG**ACGACTACAG**ATGAARACMTTTCYCATC-3’ |
| Alphagli454F19 | 5’-CCATCTCATCCCTGCGTGTCTCCGACTCAG**CGTAGACTAG**ATGAARACMTTTCYCATC-3’ |
| Alphagli454F20 | 5’-CCATCTCATCCCTGCGTGTCTCCGACTCAG**TACGAGTATG**ATGAARACMTTTCYCATC-3’ |
| Alphagli454F21 | 5’-CCATCTCATCCCTGCGTGTCTCCGACTCAG**TACTCTCGTG**ATGAARACMTTTCYCATC-3’ |
| Alphagli454F22 | 5’-CCATCTCATCCCTGCGTGTCTCCGACTCAG**TAGAGACGAG**ATGAARACMTTTCYCATC-3’ |
| Alphagli454F23 | 5’-CCATCTCATCCCTGCGTGTCTCCGACTCAG**TCGTCGCTCG**ATGAARACMTTTCYCATC-3’ |
| Alphagli454F24 | 5’-CCATCTCATCCCTGCGTGTCTCCGACTCAG**ACATACGCGT**ATGAARACMTTTCYCATC-3’ |
| Alphagli454R01 | 5’-CCTATCCCCTGTGTGCCTTGGCAGTCTCAGCTGCTGCTGTGAAATTRGWT-3’ |

**table S5:** **Fusion Primers for 454 sequencing of alpha-gliadins**. Fusion primers contain sequences needed for 454 sequencing and an gene specific part (underlined). A 10 bp ID sequence (in bold) that enabled the identification of sequences from a particular sample was present in the forward primers. The products of three amplifications were pooled together and used as a template for a second PCR amplification event using these fusion primers.
